# Bacteriophages of *Mycobacterium tuberculosis*, their diversity, and potential therapeutic uses: a review

**DOI:** 10.1186/s12879-022-07944-9

**Published:** 2022-12-22

**Authors:** Fatemeh Zeynali kelishomi, Susan Khanjani, Fatemeh Fardsanei, Hediyeh Saghi Sarabi, Farhad Nikkhahi, Behzad Dehghani

**Affiliations:** 1 Medical Microbiology Research Center, Qazvin University of Medical Sciences, Qazvin, Iran; 2 Department of Bacteriology-Virology, Shiraz University of Medical Sciences, Shiraz, Iran

**Keywords:** *Mycobacterium tuberculosis*, Drug resistance, Mycobacteriophages, Phage therapy

## Abstract

Tuberculosis (TB) caused by *Mycobacterium tuberculosis* (*M. tuberculosis*) is a highly infectious disease and worldwide health problem. Based on the WHO TB report, 9 million active TB cases are emerging, leading to 2 million deaths each year. The recent emergence of multidrug-resistant tuberculosis (MDR-TB) and extensively drug-resistant tuberculosis (XDR-TB) strains emphasizes the necessity to improve novel therapeutic plans. Among the various developing antibacterial approaches, phage therapy is thought to be a precise hopeful resolution. Mycobacteriophages are viruses that infect bacteria such as *Mycobacterium* spp., containing the *M. tuberculosis* complex. Phages and phage-derived proteins can act as promising antimicrobial agents. Also, phage cocktails can broaden the spectrum of lysis activity against bacteria. Recent researches have also shown the effective combination of antibiotics and phages to defeat the infective bacteria. There are limitations and concerns about phage therapy. For example, human immune response to phage therapy, transferring antibiotic resistance genes, emerging resistance to phages, and safety issues. So, in the present study, we introduced mycobacteriophages, their use as therapeutic agents, and their advantages and limitations as therapeutic applications.

## Background

Tuberculosis (TB) caused by *Mycobacterium tuberculosis* (*M. tuberculosis*) is a highly infectious disease and worldwide health problem, with a high mortality rate and nearly ~ 1.6 million recognized deaths in 2021. It has harmed humankind for approximately 9000 years, with the first report dating back more than 3000 years ago in India and China [1, 2]. TB had a notable effect on social health owing to decreased influence and a more negligible therapeutic effect with mycobacterial therapy. The quick prevalence of disease and the warning development of drug resistance, particularly the appearance of multidrug-resistant tuberculosis (MDR-TB) and extensively drug-resistant tuberculosis (XDR-TB) strains, have called the alarm to gain novel effective drugs; thus, finding a substitute line for the controlling and management of TB has become essential. One of the main features of *Mycobacterium* is that it produces highly resistant mutants under selective pressure conditions caused by antibiotics. The following evolutionary achievement of resistant mutants depends mostly on the mutant’s resistance rate and ability and selective elimination owing to antibiotic therapy. Among the various developing antibacterial approaches, phage therapy is thought to be a precise hopeful resolution. Bacteriophages (phages) are a type of viruses that infect bacteria and are very widespread in the environment. Bacteriophages can be used clinically to handle bacterial disease as natural antibacterial agents [3–6]. In this review, we will introduce mycobacteriophages, their use as a therapeutic approach and diagnosis tools, and their superiority and some challenges and limitations as therapeutic applications.

## Search strategies

The main literature search for published research evaluating phage therapy of *Mycobacterium tuberculosis* from 2000 to 2022 was done using the PubMed and Scopus databases.

## Inclusion and exclusion criteria

The review included full-text articles published in different countries over the last 22 years were explained the therapeutic uses of mycobacteriophages in Drug-resistant TB. The review similarly excluded articles that had not been considered by academic counterparts and articles published past the chosen period.

## *Mycobacterium tuberculosis* drug resistance

It is evaluated that drug-resistant strains of Mycobacterium will kill more than 75 million people in the next 35 years. According to the World Health Organization Global TB report, Tuberculosis mortality has increased since the COVID-19 pandemic. The Covid-19 pandemic may increase the number of new cases of tuberculosis due to resource constraints and other constraints in TB native areas [7]. Over the last two decades, multi-drug strains (MDR), extensively-drug (XDR), extremely-drug (XXDR), and total-drug resistant (TDR) strains of *M. tuberculosis* have emerged as a worldwide challenge. One of the main reasons for the prosperity of *M. tuberculosis* in causing infection and escaping the host immune response is its specific cell envelope, which is mainly composed of lipids and carbohydrates. The presence of these compounds enables the bacterium to adapt to different environmental conditions and protect the bacterium in the presence of drugs [8, 9].

For this reason, in the treatment of tuberculosis, monotherapy is not recommended, and several antibiotics use simultaneously. Standard treatment for susceptible strains of *M. tuberculosis* includes treatment with four first-line drugs (treatment with isoniazid, rifampicin, ethambutol, and pyrazinamide for 2 months, followed by treatment with isoniazid and rifampicin for 4 months) [10]. However, various factors have led to the spread of drug-resistant strains of *M. tuberculosis*. These factors include incorrect prescription of drugs, insufficient access to drugs, and poor commitment to treatment [11]. MDR-TB strains are resistant to at least two first-line drugs and should treat for 9–20 months. However, the treatment success rate of these strains is 56% compared to sensitive strains. In addition to being resistant to isoniazid and rifampicin, XDR-TB strains are resistant to fluoroquinolone and one of three second-line injectable drugs (amikacin, capreomycin, or kanamycin). Also, the success rate of treating these strains is 39% [10]. XDR-TB strains are resistant to all first- and second-line antibiotics. In recent years, new antibiotics including bedaquiline have been added to the tuberculosis treatment program. The use of bedaquiline in patients with MDR/XDR TB was able to cure 82% of patients. The recovery rate of patients with MDR TB was 89.9%, but the recovery rate of patients with XDR TB was 71.9% [12]. However, in recent years, in countries such as Iran, Italy, and India, strains resistant to all antibiotics (even resistant to antibiotics under discovery and development) have been reported [13–15], which the WHO called Drug-resistant TB (TDR-TB). Therefore, due to the emergence of these resistant and incurable strains with available antibiotics, researchers are looking to discover new drugs and even newer methods for treating tuberculosis [16].

## Risk factors for *Mycobacterium tuberculosis* infections

The main risk factors include contact with people who are infected by tuberculosis, living with people infected by human immunodeficiency virus (HIV), HIV co-infection, being a former prisoner, being a smoker, alcoholism, being an immigrant, being male, being middle-aged, health care staff and those with chronic obstructive pulmonary disease (COPD). Hospital-acquired tuberculosis infection is more commonly recognized in High-frequency TB and low and medium-income countries which report 87% annually. The number of TB cases per 100,000 Healthcare workers in some down and average-income countries is further than twofold the frequency level among the general population, and healthcare services are a significant origin of TB transfer in these countries. Another factor involved in the development of TB is urbanization, mostly among high-capacity regions. For many reasons, such as overpopulation, quick improvement, and other environmental features, many cases happened. Close contact and inhalation in the same nearby environs among patients with TB and susceptible persons cause the extent of TB. Numerous studies have revealed that TB patients need reception to the ICU conveys a high mortality level of 25–63% [17, 18]. Delays in treatment or diagnosis could lead to acute disease and higher mortality rates. Several studies have assessed risk factors for death in the treatment of TB. For example, age, sex, bacteriological case, immune and dietary condition of the host, and drug abuse, have been recognized. Effective treatment of TB is essential to treating the patient and decreasing the spread of *M. tuberculosis* in public places. But an important subject is the widespread occurrence of drug-resistant TB (DR-TB, worries around DR-TB are growing in current years. At least 5% of whole universal cases of TB have several types of drug resistance, that is, resistance to, as a minimum, one first-line anti-TB drug [19–22].

## An introduction to bacteriophages

**Fig. 1:**
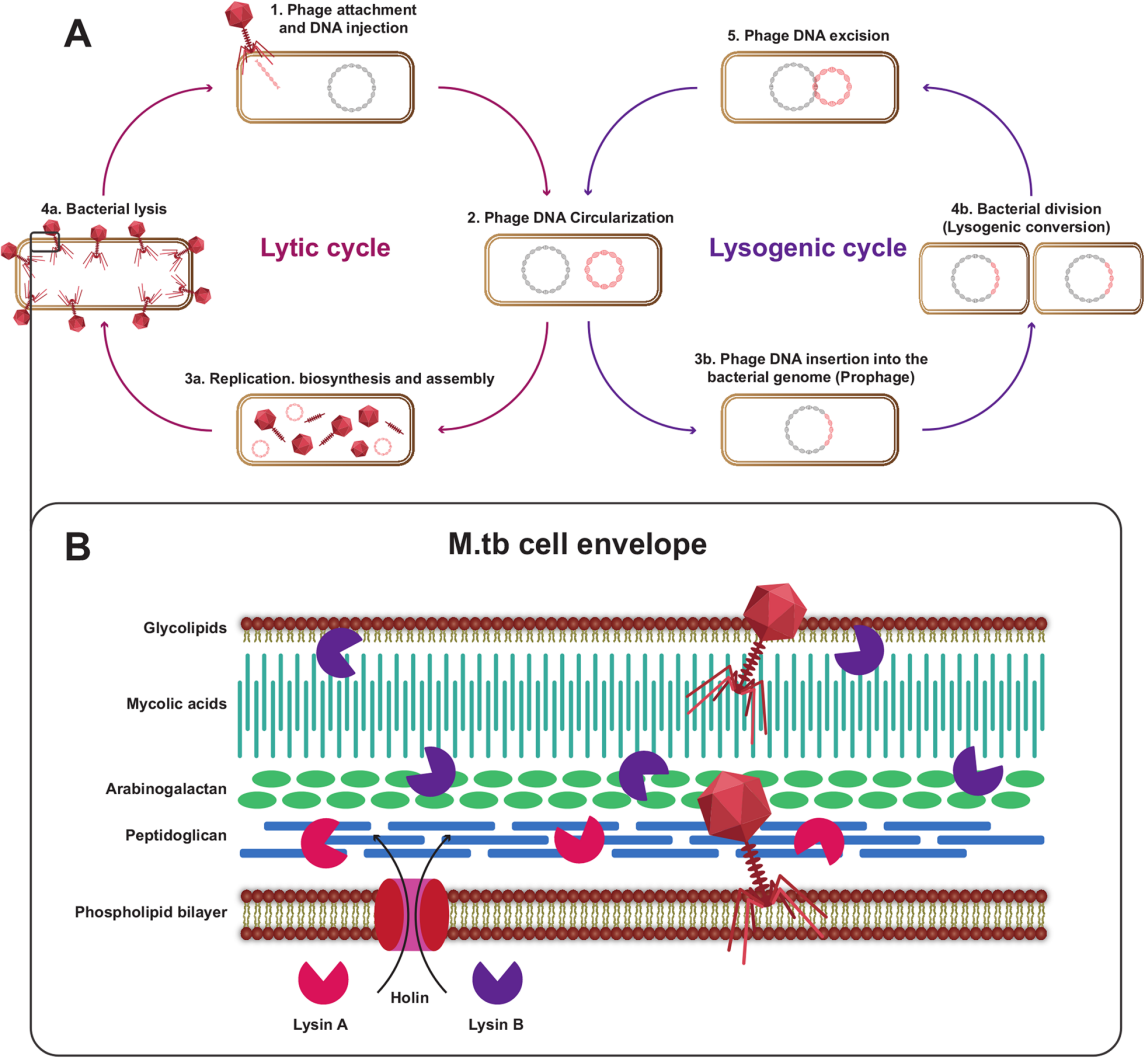
Different mechanisms of bacterial lysis by phages. **A** Lytic and lysogenic cycles of mycobacteriophages in *M. tuberculosis*. **A**1 The phage binds to *M. tuberculosis* using specific receptors and injects its genetic materials, **A**2 Phage DNA circularization occurs within the *M. tuberculosis*. Then, for particular reasons, the phage enters a lytic or a lysogenic cycle. In the case of the lytic cycle, **A**3a new phage proteins and DNA are produced and brought together in new viral elements. **A**4a The *M. tuberculosis* cells are lysed, and new viral particles are released. During the lysogenic cycle, **A**3b the phage genetic material is combined with the *M. tuberculosis* genome, and a prophage is produced. **A**4b The prophage will reproduce alongside the *M. tuberculosis* genome and will be transferred to the progeny that will gain new features. **A**5 In certain situations, the prophage genome will be cut from the bacterial genome, and the lytic cycle will be performed. **B** In most cases, mycobacteriophages lyse their host using the endolysin–holin systems. Holins act as membrane proteins to support and displace the lysins to attain their targets. Lysin A destroys the peptidoglycan, while Lysin B cuts the ester bonds between mycolic acids and the arabinogalactan to damage trehalose dimycolates (TDMs)

Bacteriophages, the most plenty organisms on the earth, are the shady subject of the biological world, making an enormous, extremely old, dynamic, and genetically various population. About 10^31^ tailed phage elements join in about 10^23^ infections per second worldwide, with the total population changing in a short time. Shortly after Felix d'Herelle discovered bacteriophage, the idea of using phage to treat the infectious disease was introduced [23–25]. In the 1940s, the use of phage therapy was restrained by the preface of penicillin and other antibiotics. While the concept of phage therapy has been about for approximately a century, it is yet well-thought-out empiric therapy in Western countries and has not been permitted for human practice so far. Although, the emergence of drug-resistant bacteria as MDR-TB and XDR-TB phage therapy is well thought-out to be a significant candidate for substitute therapeutic agents [26, 27]. According to their survival life strategies, phages display three diverse life cycles: lytic, lysogenic, and pseudo-lysogenic when infecting a bacterial host. The phage must first attach to the host cell and then inject its genetic material into the cell (Fig. 1A). A significant dissimilarity among viruses that infect bacteria and eukaryotic cells is the early procedures related to infection. Usually, bacteriophages are challenged by attaching and penetrating a bacterial cell wall. Then, expel their genomic materials the inside of the cell. The intrusion into the bacterial wall is usually related to a cell wall digesting enzyme(s), regularly mentioned as a peptidoglycan hydrolase or endolysin, often found in a tail construction of tailed phages. The phage multiplies during the lytic phase, and progeny phages explode the cell and exit. The phage does not reproduce in the lysogenic cycle, but its genome goes into a quiet state and is generally integrated into the host genome (Fig. 1A). In the pseudo lysogenic step, the phage does not experience lysogeny, nor does it display a lytic cycle, but it stays in a non-active condition. Phages that reproduce through the lytic cycle are called virulent phages, whereas those that replicate via both lytic and lysogenic cycles are identified as temperate phages. The lysogenic stage may be constant for numerous generations, and the bacteriophage could modify the phenotype of the bacterium by gene expression that is not light in the normal period of infection in a procedure identified as lysogenic conversion (Fig. 1A). Phages may have a pseudolysogeny stage in their life cycle. It refers to a condition that a phage has joined a bacterial cell and does not unify in a constant style, then will remain in this manner while situations fall out which trigger them to go into the lytic or lysogenic life cycle. The carrier state defines combinations of bacteria and of bacteriophages that are invariable and stable. A section of bacteria is persistent, but some sensitive alternates’ attendance seems to endure the phage population so that both progress [28–32].

## Phage characterization

Phages can be isolated from different sources including soil, water, and sewage [3]. The Mycobacteriophage was primarily isolated in 1954. Through the 1960s and 1970s, phages were used for typing *M. tuberculosis* clinical isolate in epidemiological research [33]. Most of the lytic phages of *M. tuberculosis* belong to Order—*Caudovirales*; the Family—*Siphoviridae*, and cluster K [34, 35], which is divided into seven sub-clusters (Table 1). Due to the massive genetic multiplicity between bacteriophages, they are classified into clusters and subclusters. Cluster K is one of the known clusters in which all members can lyse *M. tuberculosis*, and Mycobacteriophage DS6A infects the *M. tuberculosis complex. Siphoviridae* has a high flexible tail structure that makes it difficult to identify. The pointed capsid layer is occupied with dsDNA. The genomes of Cluster K phage (average genome length, 60 kbp) have some uncommon structures containing start-associated sequences (SAS) and extended start-associated sequences (ESAS). It is believed that the discovery of new mycobacteriophages would help grow the present database and can be conducted to recognition of un-explored infectious phages as a source of hydrolytic enzymes, for example, Endolysins, EPS depolymerase, and Phospholipases/Esterases [33, 36, 37].

**Table 1:** Cluster K mycobacteriophages and their characteristics (Retrieved from Phagesdb.org and Nucleotide, GenBank)

Phage	Subcluster	Family	RefSeq/GenBank Accession no.	Genome size (bp)	Genome type
ActinUp	K1	*Siphoviridae*	MH051246	59,812	DNA linear
Adephagia	K1	*Siphoviridae*	JF704105	59,646	DNA linear
Adonis	K1	*Siphoviridae*	MH001453	60,031	DNA linear
AlishaPH	K1	*Siphoviridae*	MH077577	57,034	DNA linear
AlleyCat	K5	*Siphoviridae*	MF185717	62,112	DNA linear
Amelie	K1	*Siphoviridae*	KX808132	56,439	DNA linear
Amgine	K6	*Siphoviridae*	MF324915	62,236	DNA linear
Aminay	K7	*Siphoviridae*	MH509442	60,430	DNA linear
Amohnition	K6	*Siphoviridae*	MF140398	61,761	DNA linear
Anaya	K1	*Siphoviridae*	JF704106	60,835	DNA linear
Angelica	K1	*Siphoviridae*	HM152764	59,598	DNA linear
Apocalypse	K1	*Siphoviridae*	MF668267	59,947	DNA linear
Asayake	K1	*Siphoviridae*	MW712723	59,905	DNA linear
Atiba	K1	*Siphoviridae*	MN234230	59,556	DNA linear
BaghKamala	K1	*Siphoviridae*	MW712730	59,132	DNA linear
BarrelRoll	K1	*Siphoviridae*	JN643714	59,672	DNA linear
BEEST	K1	*Siphoviridae*	MH509444	59,906	DNA linear
Beezoo	K1	*Siphoviridae*	MH371113	60,494	DNA linear
Bella96	K1	*Siphoviridae*	MF377440	60,746	DNA linear
Belladonna	K1	*Siphoviridae*	MH697578	59,708	DNA linear
Biglebops	K1	*Siphoviridae*	MH399770	56,454	DNA linear
Blizzard	K1	*Siphoviridae*	MW712733	59,905	DNA linear
Boiiii	K1	*Siphoviridae*	OK310505	59,907	DNA linear
Boilgate	K4	*Siphoviridae*	MZ274310	57,889	DNA linear
BoostSeason	K2	*Siphoviridae*	MH834601	58,078	DNA linear
Bryler	K6	*Siphoviridae*	MN369762	57,666	DNA linear
Cain	K6	*Siphoviridae*	MF324913	60,813	DNA linear
Capricorn	K1	*Siphoviridae*	MK112537	59,708	DNA linear
CaseJules	K1	*Siphoviridae*	OK040784	59,905	DNA linear
Chancellor	K4	*Siphoviridae*	MF140402	57,697	DNA linear
Cheetobro	K4	*Siphoviridae*	KJ944841	57,253	DNA linear
Chris	K1	*Siphoviridae*	MT310860	62,067	DNA linear
Collard	K5	*Siphoviridae*	MH651171	61,395	DNA linear
Crew	K1	*Siphoviridae*	KY380102	59,707	DNA linear
CrimD	K1	*Siphoviridae*	HM152767	59,798	DNA linear
Curiosium	K1	*Siphoviridae*	MN234226	61,222	DNA linear
Dalmuri	K1	*Siphoviridae*	MH727544	59,708	DNA linear
DarthP	K6	*Siphoviridae*	MF140406	61,594	DNA Linear
Deby	K1	*Siphoviridae*	MG962364	60,463	DNA linear
Devera	K1	*Siphoviridae*	OK040778	60,618	DNA linear
DismalFunk	K2	*Siphoviridae*	MF140408	58,129	DNA linear
DismalStressor	K2	*Siphoviridae*	MH727545	58,129	DNA linear
Dole	K1	*Siphoviridae*	MZ005674	60,621	DNA linear
DrHayes	K1	*Siphoviridae*	KX657795	60,526	DNA linear
DS6A	Singleton	*Siphoviridae*	JN698994	60,588	DNA linear
Durfee	K1	*Siphoviridae*	MW712734	59,905	DNA linear
Edugator	K5	*Siphoviridae*	MF185719	63,344	DNA linear
Efra2	K1	*Siphoviridae*	MN234174	61,284	DNA linear
Ekdilam	K6	*Siphoviridae*	MN234199	61,772	DNA linear
Ellie	K6	*Siphoviridae*	MT723940	61,945	DNA linear
Emerson	K1	*Siphoviridae*	KJ567045	60,310	DNA linear
Enkosi	K1	*Siphoviridae*	KT281789	59,052	DNA linear
Eponine	K4	*Siphoviridae*	MN945904	58,678	DNA linear
Fefferhead	K6	*Siphoviridae*	MW601222	61,366	DNA linear
Findley	K2	*Siphoviridae*	MF140411	58,150	DNA linear
Fionnbharth	K4	*Siphoviridae*	JN831653	58,076	DNA linear
Ganymede	K1	*Siphoviridae*	ON081331	59,719	DNA linear
Gengar	K5	*Siphoviridae*	KX636165	61,626	DNA linear
Geralini	K1	*Siphoviridae*	MN234182	59,818	DNA linear
Guanica15	K1	*Siphoviridae*	MN234201	60,974	DNA linear
Guilsminger	K5	*Siphoviridae*	MF185720	63,153	DNA linear
Hammy	K6	*Siphoviridae*	KY087993	61,812	DNA linear
HedwigODU	K1	*Siphoviridae*	KX585253	59,812	DNA linear
Homura	K1	*Siphoviridae*	MH536821	59,708	DNA linear
Hurricane	K3	*Siphoviridae*	MF373841	61,318	DNA linear
Hyperbowlee	K1	*Siphoviridae*	OM818330	59,905	DNA linear
Illumine	K1	*Siphoviridae*	OK040782	60,620	DNA linear
Inky	K1	*Siphoviridae*	MN369746	59,708	DNA linear
InvictusManeo	K5	*Siphoviridae*	MZ958747	61,147	DNA linear
Jarvi	K1	*Siphoviridae*	MW862985	59,708	DNA linear
JAWS	K1	*Siphoviridae*	JN185608	59,749	DNA linear
Jecky11	K1	*Siphoviridae*	MF140412	59,708	DNA linear
JF1	K4	*Siphoviridae*	MT310882	57,990	DNA linear
Joy99	K1	*Siphoviridae*	MH536822	59,837	DNA linear
Juliette	K4	*Siphoviridae*	MW601218	58,071	DNA linear
Keshu	K3	*Siphoviridae*	KP027199	61,251	DNA linear
KiSi	K1	*Siphoviridae*	MK376955	62,558	DNA linear
Kratio	K5	*Siphoviridae*	KM923971	62,738	DNA linear
Krueger	K6	*Siphoviridae*	MF324914	60,321	DNA linear
Larva	K5	*Siphoviridae*	JN243855	62,991	DNA linear
LastHope	K1	*Siphoviridae*	MF140416	60,934	DNA linear
LaterM	K1	*Siphoviridae*	MG962371	60,143	DNA linear
LeMond	K1	*Siphoviridae*	MH910038	62,515	DNA linear
Leston	K5	*Siphoviridae*	MH051255	61,808	DNA linear
LilPharaoh	K1	*Siphoviridae*	MF919518	56,167	DNA linear
Lind NT	K1	*Siphoviridae*	KX641264	60,053	DNA linear
MacCheese	K3	*Siphoviridae*	JX042579	61,567	DNA linear
Malthus	K4	*Siphoviridae*	MN369761	57,802	DNA linear
Macroliusprime	K2	*Siphoviridae*	KX688047	58,129	DNA linear
MarkPhew	K1	*Siphoviridae*	MT310859	62,153	DNA linear
Marshawn	K6	*Siphoviridae*	MN284895	61,464	DNA linear
Mdavu	K1	*Siphoviridae*	MN586025	56,443	DNA linear
MeaningOfLife	K1	*Siphoviridae*	MW862984	60,432	DNA linear
Milly	K2	*Siphoviridae*	KP027206	58,211	DNA linear
MissDaisy	K4	*Siphoviridae*	MK524485	54,464	DNA linear
Mitti	K4	*Siphoviridae*	KY087992	57,895	DNA linear
Mufasa	K2	*Siphoviridae*	KT591490	58,065	DNA linear
Murucutumbu	K1	*Siphoviridae*	KM677211	60,609	DNA linear
Mynx	K1	*Siphoviridae*	MH513977	60,055	DNA linear
Nibb	K1	*Siphoviridae*	MK460246	62,293	DNA linear
Nikao	K1	*Siphoviridae*	OP297530	59,052	DNA linear
Niklas	K1	*Siphoviridae*	MK494119	60,989	DNA linear
Nutello	K1	*Siphoviridae*	OM913583	56,439	DNA linear
OkiRoe	K5	*Siphoviridae*	KJ567042	62,661	DNA linear
Omnicron	K5	*Siphoviridae*	KM363596	61,511	DNA linear
Oscar	K1	*Siphoviridae*	MH910039	62,437	DNA linear
Padfoot	K1	*Siphoviridae*	MW862990	59,905	DNA linear
Padpat	K1	*Siphoviridae*	ON724013	60,310	DNA linear
Paola	K5	*Siphoviridae*	MG962374	61,535	DNA linear
Patt	K4	*Siphoviridae*	MK524488	54,611	DNA linear
Peanam	K1	*Siphoviridae*	MF185722	61,041	DNA linear
Peel	K1	*Siphoviridae*	MW862979	59,711	DNA linear
PhelpsODU	K6	*Siphoviridae*	MF324909	56,580	DNA linear
Phrank	K6	*Siphoviridae*	MF324912	61,109	DNA linear
Piatt	K1	*Siphoviridae*	OM913584	59,905	DNA linear
Pixie	K3	*Siphoviridae*	JF937104	61,147	DNA linear
Pokerus	K1	*Siphoviridae*	ON081329	59,775	DNA linear
Prithvi	K1	*Siphoviridae*	MK016503	60,311	DNA linear
Psycho	K5	*Siphoviridae*	MW435854	62,110	DNA linear
QuincyRose	K1	*Siphoviridae*	MZ648037	59,719	DNA linear
Ramen	K1	*Siphoviridae*	MN234197	59,462	DNA linear
Rando14	K5	*Siphoviridae*	MH697592	59,925	DNA linear
Rapunzel97	K1	*Siphoviridae*	MN234231	59,687	DNA linear
Reptar3000	K4	*Siphoviridae*	MH926058	54,601	DNA linear
Ruthiejr	K4	*Siphoviridae*	ON526978	57,858	DNA linear
SamScheppers	K4	*Siphoviridae*	MH051258	58,351	DNA linear
SamuelLPlaqson	K1	*Siphoviridae*	KX657794	60,526	DNA linear
Scarlett	K1	*Siphoviridae*	MH910042	62,306	DNA linear
SgtBeansprout	K1	*Siphoviridae*	MH020245	56,439	DNA linear
Shaobing	K1	*Siphoviridae*	MK310138	61,030	DNA linear
SehdLockHolmes	K3	*Siphoviridae*	KR080206	61,081	DNA linear
ShiaSurprise	K1	*Siphoviridae*	ON260816	59,905	DNA linear
SirPhilip	K6	*Siphoviridae*	MF324911	61,882	DNA linear
Slarp	K4	*Siphoviridae*	KT361920	57,256	DNA linear
Slimphazie	K1	*Siphoviridae*	MF140428	60,143	DNA linear
SoSeph	K5	*Siphoviridae*	MZ322016	61,968	DNA linear
Spock	K1	*Siphoviridae*	MN369742	59,709	DNA linear
Stinson	K1	*Siphoviridae*	MZ355721	59,918	DNA linear
Sully	K1	*Siphoviridae*	MF919532	59,873	DNA linear
Tachez	K1	*Siphoviridae*	MF140430	59,556	DNA linear
Taquito	K4	*Siphoviridae*	KX621007	58,390	DNA linear
TBond007	K3	*Siphoviridae*	KX683428	61,145	DNA linear
Thyatira	K5	*Siphoviridae*	MH576966	63,874	DNA linear
Tiri	K1	*Siphoviridae*	ON526984	59,449	DNA linear
TM4	K2	*Siphoviridae*	AF068845	52,797	DNA linear
TreyKay	K1	*Siphoviridae*	MF472892	60,311	DNA linear
Twitch	K1	*Siphoviridae*	MW712722	59,711	DNA linear
Unicorn	K6	*Siphoviridae*	MF324908	61,208	DNA linear
Urkel	K1	*Siphoviridae*	KX657796	60,526	DNA linear
Validus	K1	*Siphoviridae*	KF713486	62,466	DNA linear
Veliki	K1	*Siphoviridae*	MN234205	59,734	DNA linear
Waterfoul	K5	*Siphoviridae*	KX585251	61,248	DNA linear
Wintermute	K4	*Siphoviridae*	MF140435	58,046	DNA linear
Ximenita	K6	*Siphoviridae*	MN945901	61,027	DNA linear
YoureAdopted	K1	*Siphoviridae*	MK460247	59,504	DNA linear
Yuna	K6	*Siphoviridae*	MN234176	62,192	DNA linear
Yunkelll	K1	*Siphoviridae*	MN234165	60,757	DNA linear
Zavala	K1	*Siphoviridae*	MN234198	59,969	DNA linear
ZoeJ	K2	*Siphoviridae*	KJ510412	57,315	DNA linear

## Phage proteins

In the last phase of the lytic cycle, newly assembled phage particles must be released from the infected host. Therefore, the phage must destroy peptidoglycan, mycolic acid, and cell membrane structures. Phage produces two proteins to release from the cytoplasm of host bacteria: endolysin and holin. Endolysins cut covalent links in the peptidoglycan (PG) and disrupt cell wall integrity that supports the discharge of phage particles from the bacterial host [1–3].

## Holin

Holins are a large group of small hydrophobic membrane proteins holding a transmembrane area congested in the inner membrane and cause cell membrane permeability by making perforations that collapse the proton motive force (PMF) of the cell membrane and leading to cell death (Fig. 1B). Holins are thought-out to be the simplest biological device as they control the secreted and availability of endolysins to the cell wall. Holins do a different well-known function, which contains release of gene transferal mediators, having a function in biofilm development, simplifying several procedures essential for differentiation, such as spore germination, [4] aid in diverse responses to stressful situations [5] and release toxins and associated proteins [38–42].

## Endolysin

Mycobacterial envelop contains a cytoplasmic membrane, a peptidoglycan layer covalently connected to the arabinogalactan-peptidoglycan complex, and mycolic acids (Fig. 1B). Mycobacteriophages are the phages that infect mycobacterial species. Mycobacteriophages produce two endolysins, LysinA and LysinB, for overcoming these complex layers. LysinA and LysinB affect peptidoglycan and mycolic acid arabinogalactan separately. LysB is a mycolylarabinogalactan esterase that cuts the ester link among arabinogalactan and mycolic acid (Fig. 1B). Thus, cell lysis occurs following the loss of communication between the Mycobacterium cell wall and the outer membrane. Although a great number of mycobacteriophages have been identified, few studies have been performed on mycobacteriophage endolysins. Most studies have been reported about endolysin of D29 and Ms6 mycobacteriophages. Fraga et al. showed for the first time that recombinant LysB exhibits lytic activity on *M. ulcerans* isolates [43]. Also, they showed that using LysB for the management of mouse models of *M. ulcerans* footpad infection inhibits cell proliferation. Pohane et al. carried out a study on the structure and function of the Lysin A of Mycobacteriophage D29. By making several structures, they studied the details of LysinA and obtained the shortest protein sequence with a catalytic domain [44]. Mycobacteriophage lysines are considered a potential alternative treatment for mycobacterial infections caused by MDR and XDR strains [5, 45, 46].

## Specificity and host range

**Fig. 2:**
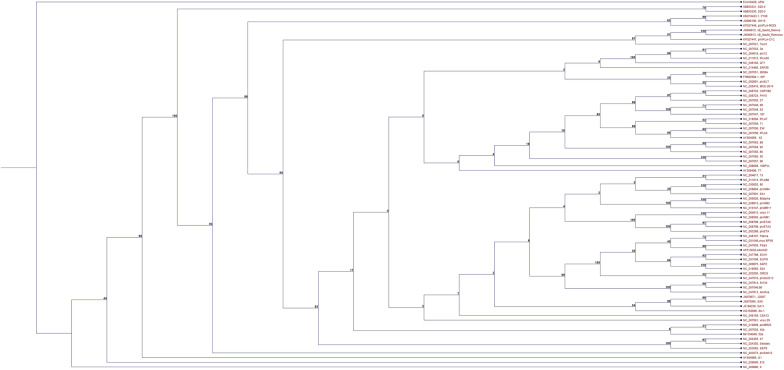
The phylogenetic tree of 159 mycobacteriophages from the order *Caudovirales*, Family *Siphoviridae*, which are listed in (Table 1). This analysis was performed using the neighbor-joining method (bootstrap: 1000). Generally, the majority of node bootstrap scores were above 70%; therefore, the quality of the tree was satisfying. The figure shows two large clades, which upper one contains a small number of sequences. The lower large clade is divided into two clusters (upper and lower); the lower one contains the majority of sequences

The host range of a bacteriophage is specified as the extent of hosts that it can contaminate. This span is related to host features (e.g., protection and restriction-modification systems and the existence of phage receptors), environmental elements (e.g., temperature and pH), and structures determined by the phage. Specific bacteriophages frequently exhibit a narrow host range and contaminate a narrow spectrum of bacterial strains of similar species. In comparison, common bacteriophages intrinsically exhibit a wide host range. Phage–host interaction is unique, and phages are very precise to their bacterial hosts, and they replicate using the facilities of the host cell. The first stage in the bacteriophage life cycle is its binding to the bacterial cell surface by a receptor on the phage tail or capsid. The capability of a phage to recognize and bind to receptors is one of the factors that influence its host range. Various mycobacteriophages belonging to cluster K (Table 1) can infect a different range of hosts, including slow-growing mycobacteria (e.g., *M. tuberculosis*) and fast-growing (e.g., *M. smegmatis*), however comprehensive visions into specific host ranges stay mostly missing because of the aspect that the most common identified mycobacteriophages were isolated via *M. smegmatis* mc2155. It is commonly well-thought-out, in the situation of their therapeutic usage, that lytic phages by a wide host range (e.g., at genus or species level) are more helpful in fighting bacterial infection than those with a narrow host range (e.g., at strain level) [25, 47, 48].A phylogenic tree of All 159 mycobacteriophage according to Whole-genome sequencing available in Genebank database were analysed using neighbor-joining method. The figure shows that two large clades, which upper one contains a small number of sequence. the lower large clade is divided into two cluster (Fig. 2).

## Mycobacteriophages as diagnostics

TB control is confined by present detection methods. Clinicians use X-rays, microscopy, and cultures as widespread implements to identify TB. Using molecular methods such as the GeneXpert system, TB is detected in a short time and with high sensitivity, but so far, this device has not been widely used. Culturing of *Mycobacterium* is known as the gold standard diagnostic, but many *mycobacterial* species are slow-growing, such as *M. tuberculosis* and *M. bovis*. Phage-based diagnosis generally comprised of two overall extents: phage amplified biologically (PhAB) assay and phage reporter assays (PRAs). The PhAB uses a definite characteristic of the phage’s natural capacity to infect, strengthen, and disrupt the cells to identify the mycobacteria. PRAs usually encompass genetically altered bacteriophages or their hosts with the aim of a fluorescent, luminescent or different signal can be identified. Previously, phage-based kits existed and were mainly considered for *M. tuberculosis* recognition in human sputum samples. Nowadays, it is probable to use an in-house alternative test, which founds a laboratory-established phage amplification assay (PA) not expressively diverse from the commercial one. This might characterize an appropriate substitute for PCR tests, particularly in low-income countries, because it depends on only simple microbiological methods. The defect of PA can be an ineffective infection in a significant number of bacteria in the specimen, which can limit from half to four-fifths of the measured CFU, and could be triggered by some reasons; e.g., phage replication does not happen in dormant bacteria [49, 50].

## In vivo experimentation

**Fig. 3:**
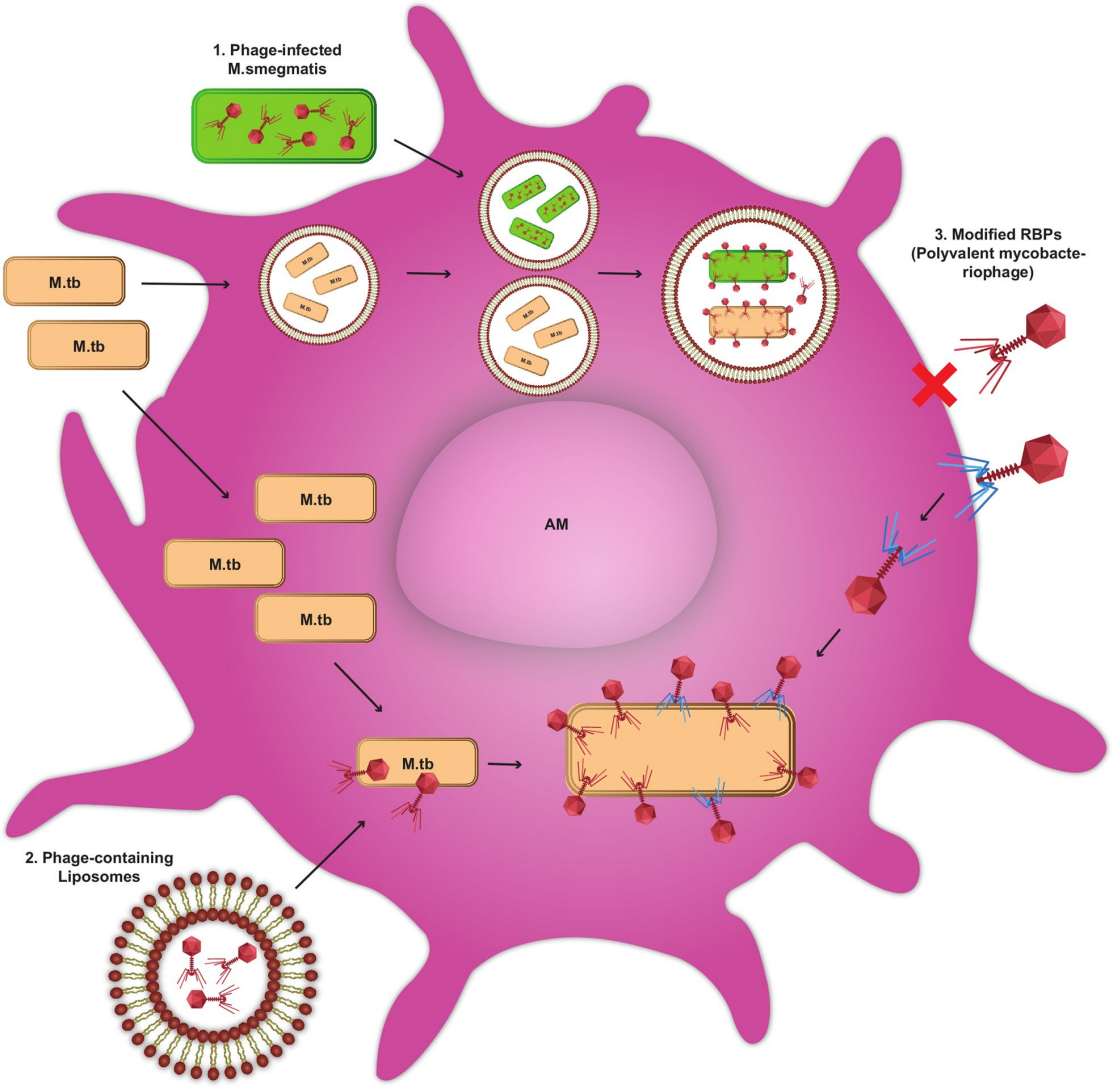
Different plans can be applied to transfer mycobacteriophages into mammalian cells to achieve *M. tuberculosis*: (1) The *M. smegmatis* infected by Mycobacteriophage doing such as carriers are phagocytized via alveolar macrophages (AMs), conveyed into phagosomes containing *M. tuberculosis*. Mycobacteriophages replicate into *M. smegmatis,* lyse it, and access the phagosome lumen, then infect and lyse *M. tuberculosis*. (2) Liposome-linked mycobacteriophages are more efficient in infecting mammalian cells than free phages. (3) Production of polyvalent mycobacteriophages to identify eukaryote cells additionally the *M. tuberculosis* cell surface receptors

After in vitro examinations, each new treatment candidate must be evaluated for efficacy and safety in an animal model and then performed in human experiments. Each TB therapy choice will requirement to overwhelming defies the infection plans (tissue/granuloma diffusion, penetration to host cell, drug interface with HIV treatment). Moreover, they must be rare in toxicity and confrontational properties on microflora, short in time, and will have to be made accessible in the countryside and poor regions [51]. The key benefits of phage therapy are low charge of manufacturing, no side effects on microflora, and auto-adjustment of phage levels in the patient. The negative impacts of phage and chemical medicines have not been recognized. The phages could not entirely remove a bacterial pathogen alone because they would lose the bacterial host devices. However, effective phage management could expressively decrease the number of targeted bacteria. Finally, the mammalian immune system entirely removes pathogen remains from the tissue. In this procedure called “Immunophage Synergy”, the act of the immune system is required and counterparts the phage antimicrobial activity, seen in neutrophil-phage collaboration [52]. Moreover, phages have the potential to stimulate anti-inflammatory cytokines over their contact with host immune cells, helping to decrease inflammation and tissue injury. For example, bacteria were used as a carrier to transport lytic phages into the macrophages of the mouse to destroy methicillin-resistant *S. aureus* inward of the cells. About *M. tuberculosis*, Mycobacteriophage sending into macrophages has been reached using *M. smegmatis* or liposomes. As well as being fast-growing and non-virulent, *M. smegmatis* can similarly render as host bacterial storage for Mycobacteriophage multiplying phage titers before attainment of the targeted *M. tuberculosis*. However, *M. smegmatis* intervened in mycobacteriophage transfer has been confirmed in vitro. Owing to its pathogenicity in mice models, it could not be a proper strain to achieve phage transport training in vivo. The high specificity of DS-6A creates it a noteworthy candidate for TB therapy. Sula et al. gained incentive results causing treatment by DS-6A and a decrease in lesions in the spleen, lungs, and livers of guinea pigs [53]. In a study by Nieth et al., a non-bacterial vector was used to send bacteriophages into infected cells. They tried to encapsulate bacteriophages into liposomes. Additionally, they showed that liposome-associated bacteriophages are driven up into eukaryotic cells more capably than free bacteriophages (Fig. 3) [54]. These are important indications in the progress of an intracellular bacteriophage therapy that may be beneficial in combat contrary to multi-drug-resistant intracellular pathogens such as *M. tuberculosis* [25, 51].

## Challenges of using mycobacteriophages in the treatment of tuberculosis

Mycobacteriophages mainly have a limited host spectrum (narrow) which can be solved by having a rich phage database and also by using bioengineering methods. Due to the intracellular nature of *M. tuberculosis*, phage access to the bacterium is difficult. However, using carriers such as *M. smegmatis* and phage encapsulation [55–58], the phage can be transported and reach the bacteria. Another challenge with mycobacteriophage therapy is phage resistance. Due to the widespread use of phages as therapeutic and ecological bio-control agents, selective pressure could lead to the expansion of resistant bacteria. Long interaction between phages and bacteria has caused bacteria to develop a variety of mechanisms to escape from phages, and in phages emerging certain approaches to escape the antiviral systems [59]. Although the appearance of resistant bacteria, phages will find an approach to confirm their dispersion. Based on data from various studies, phage resistance may be due to damage or altering their external receptors over mutation of genes accountable for the production of these receptors, so inhibiting phage incorporation, prevention of phage DNA diffusion, hindering of the receptor(s) inhibition of intracellular phage association and hydrolyses phage genome by the production of restriction endonuclease enzymes. Phage-derived enzymes can destroy cell surface receptors. So far, it was believed phage therapy, like antibiotics, just decreases the number of bacteria. But treatment breaks happen when bacteria are provided to improve phage resistance through phage management. Therefore, some strategies have been suggested to inhibit phage resistance in bacteria, including using phage cocktails instead of monotherapy and phage engineering that goes beyond simple phage monotherapy to preclude resistance, such as multi-phage cocktails, phage engineering, and combining phages with antibiotics [60, 61]. Some studies have warned of the possibility of phage toxicity to humans. But the genes with the potential for toxicity can be eliminated using genetic bioengineering techniques. It is desirable to identify all genes and protein functions before using phages in clinical trials to prevent such complications [62, 63].

## Combination therapy

The emergence of drug-resistant bacterial pathogens such as MDR-TB and XDR-TB has become an intense challenge for scientists and the health of the community. The absence of efficient therapeutic procedures for MDR-TB and XDR-TB isolates needs alternative and innovative ways. The long treatment period, side effects, and high cost in unindustrialized countries have caused unfortunate agreement regarding using treatment procedures, additional operations the occurrence of drug-resistant strains. Novel antimicrobial agents, including bedaquiline, have been progressive; however, the necessity for novel therapeutic plans is inevitable [64]. AK15, a small mycobacteriophage-derived peptide, and its isomer AK15-6 exhibit effective anti-*M. tuberculosis* activity. Both AK15 and AK15-6 directly prevented *M. tuberculosis* by membrane interruption. Also, they displayed cell selectivity and synergistic properties with rifampicin. They proficiently decreased the mycobacterial load in the lungs of mice infected by *M. tuberculosis* [65]. Carlos et al. prepared a cocktail of five phages that reduces the occurrence of phage resistance and cross-resistance and powerfully destroys the *M. tuberculosis* strains [2].

Additionally, these phages act without antagonistic effect on antibiotics and infect equally isoniazid-resistant and -sensitive strains [66]. Yeswanth et al. evaluated the effect of phage cocktails on mycobacterium growth. In their 5-phage cocktail, two of them (D29 and TM4) were identified to infect *M. tuberculosis* isolates. These two phages and DS6A were grown via *M. tuberculosis* (H37Ra) as a host. Mycobacteriophages displayed synergy with antimicrobial agents, for instance, rifampicin and isoniazid. Finally, it was determined that mycobacteriophages are effective in inhibiting *M. tuberculosis* equally in the lag and log phase for some weeks. These results have significant effects on developed phage therapy for Mycobacterium [67].

## Limitations of phage therapy

The concerns about phage therapy as antibacterial agents mostly contain safety and effectiveness subjects and an increase in a possible immune response to any ordered phage. The collected information shows gaps in our considerate clinical association between the reaction among phages and the immune system. Development optimization and purification plans of phages are additional problems required to discuss. Progresses in molecular biology and biotechnology can resolve the difficulties that humans are facing now [68, 69]. Phages have been revealed to be able to transfer genes encoded antibiotic resistance and toxins into host bacterial cells through transduction procedure. Thus, such hazardous genes should be screened through phage therapy. The main goal of phage therapy is to increase the number of phages in the bacterial hosts, which occurs by using host conveniences, but few studies have been done on the side effects of this occurrence. Moreover, Industrial manufacture is a considerable issue in the therapeutic use of phage-encoded proteins. Safety procedures are the essential worries which must be taken into attention through the production procedure [48, 70].

## Current concept and further research

Bacteriophages lyse the bacterial hosts with complex mechanisms, of which little has been studied so far. Thus, more studies are needed to understand their enzymatic machinery, regulatory methods, and biochemical properties. A typical feature of mycobacteria is their complex cell envelop required for intracellular survival. So, inhibition of its formation can be an effective manner in treating tuberculosis. The lysis enzymes produced by Mycobacteriophage appear to target the main structure of the cell envelope and seem to be hopeful candidates for spoiling mycobacteria [45, 46]. Many recent studies are investigating the potential of phage-derived LysA and LysB to kill Mycobacterium, and it has been found that purified recombinant of two enzymes will be more effective. Although the novelty and tendency to use these proteins as a substitute for antibiotics, additional investigation is still required for their medical practice of them. A substitution might be the application of mycobacteriophages prophylactically instead of therapeutic goals. For instance, family or colleagues of patients newly detected with respiratory tuberculosis can consume aspirated phages to inhibit the spread and acquirement of the illness [44, 71]. Despite the numerous advantages of phage therapy in the treatment of infectious diseases, there are obstacles regarding this treatment method. For example, we can mention the lack of regulation for this method and the lack of sufficient scientific evidence [72]. With the increase of in vitro and in vivo studies, we can learn about various aspects of mycobacteriophage therapy and the interaction between phage and the host body and immune system.

In cases where antibiotics alone cannot eliminate the infection, mycobacteriophages can be used along with antibiotics. By studying the mycobacteriophage structure and its enzymes extensively, mycobacteriophage therapy can be personalized [73], and the combination of antibiotics and personalized phage therapy can be a promising method in the treatment of drug-resistant tuberculosis.

## Conclusions

The emergence of multi-drug resistant (MDR) and extensively drug-resistant (XDR) *M. tuberculosis* strains has become a global concern. Among infectious diseases, tuberculosis has the most mortality rate and is increasing. Mycobacterium genome undergoes mutations that subsequently can avoid the drugs generally used to prevent them. The prevalence of resistant strains through the control procedures and treatment of the disease is more complicated than, mainly, when the patient is co-infected by HIV. So, efforts have begun to use an old method to treat bacterial infection that is phage therapy. Phage and Phage-derived proteins could become novel sources of antimicrobial agents. But phage therapy is relatively in its early stages and is full of complications [5, 26]. All studies linking phage management were led to a target to effective treatment of patients more than to resource indication of phage-mediated therapeutic effectiveness; thus, antimicrobial agents besides the phages are often used in their treatments. On the other hand, it is challenging to agree that phage therapy combine with antibiotics [74]. Phage cocktails can be intended to improve the range of activity extent by little active attention and improve the range of activity depth. With only chemotherapeutic combination therapies, in comparison, the main importance in the treatment particularly of special, recognized pathogens, for instance, *M. tuberculosis*, as a substitution is commonly on improving spectrum of activity profundity. So, to formulize phage cocktails to also fight the development of resistance, more consideration is necessary [75]. The development of nanomedicine has been considered a biological vehicle to perform new theranostics (therapeutics and diagnostics) programs. In current years, bacteriophage investigation notices this course, which has opened up novel paths in drug and gene transfer investigations. Phage endolysins as a new therapeutic scheme has received noteworthy consideration. So far, various endolysins are described, which display-worthy results in the treatment of antibiotic-resistant bacteria. Yet, endolysin also has some challenges. One limitation of endolysin is its limited in-vivo half-life because of the output of cytokines’ inflammatory reaction and the neutralizing antibodies in contrast to it. Novel approaches are required to improve widespread chimeric lysin, to dominate these immunological reactions against endolysin. Though endolysins are demonstrated to be helpful as new therapeutics, additional investigation is essential to study their construction and engineerability in clinical trials [76, 77].

## Data Availability

The datasets used and analyzed during the current study are available from published articles and NCBI databank.

## Abbreviations

TB: Tuberculosis
MDR-TB: Multidrug-resistant tuberculosis
XDR-TB: Extensively drug-resistant tuberculosis
XXDR: Extremely-drug
TDR: Total-drug resistant
TDR-TB: Drug-resistant TB
TB: Tuberculosis
WGS: Whole Genome Sequencing
SNPs: Single nucleotide polymorphisms
SNSs: Single nucleotide substitutions
*katG*: Catalase/peroxidase
*gyrA*: DNA Gyrase
SAS: Start-associated sequences
ESAS: Extended start-associated sequences
PhAB: Phage amplified biologically
PRAs: Assay and phage reporter assays
PA: Amplification assay
PMF: Proton motive force
